# The efficacy and safety of neoadjuvant chemoradiotherapy combined with immunotherapy for locally advanced rectal cancer patients: a systematic review

**DOI:** 10.3389/fimmu.2024.1392499

**Published:** 2024-05-15

**Authors:** Lei Yang, Xiujing Cui, Fengpeng Wu, Zifeng Chi, Linlin Xiao, Xuan Wang, Zezheng Liang, Xiaoning Li, Qiyao Yu, Xueqin Lin, Chao Gao

**Affiliations:** ^1^ Department of Radiation Oncology, The Fourth Hospital of Hebei Medical University, Shijiazhuang, China; ^2^ Department of Research, The Fourth Hospital of Hebei Medical University, Shijiazhuang, Hebei Province, China

**Keywords:** PD-1/PD-L1 inhibitors, neoadjuvant immunotherapy, rectal cancer (RC), systematic review, neoadjuvant chemoradiotherapy

## Abstract

**Background:**

Several studies have explored the effectiveness of PD-1/PD-L1 inhibitors combined with neoadjuvant chemoradiotherapy (nCRT) in the treatment of locally advanced rectal cancer(LARC), particularly in microsatellite stable(MSS) or mismatch repair proficient(pMMR) LARC patients. We undertook a single-arm systematic review to comprehensively evaluate the advantages and potential risks associated with the use of PD-1/PD-L1 inhibitors in conjunction with nCRT for patients diagnosed with locally advanced rectal cancer.

**Methods:**

The PubMed, Embase, Cochrane Library, ClinicalTrials.gov, ASCO and ESMO were searched for related studies. The main outcomes were pathologic complete response (pCR), major pathological response (MPR), anal preservation, and adverse effects (AEs).

**Results:**

Fourteen articles including 533 locally advanced rectal cancer (LARC) patients were analyzed. The pooled pCR, MPR, and anal preservation rates were 36%, 66% and 86%. Grade ≥3 adverse events occurred in 20%. Subgroup analysis showed that; dMMR/MSI-H had a pooled pCR (100%) and MPR (100%), pMMR/MSS had a pooled pCR (38%) and MPR (60%); the short-course radiotherapy and long-course radiotherapy had pooled pCR rates of 51% and 30%, respectively. The rates of pCR for the concurrent and sequential immuno-chemoradiotherapy subgroups at 30% and 40%, mirroring pCR rates for the PD-L1 and PD-1 inhibitor subgroups were 32% and 40%, respectively.

**Conclusion:**

In cases of locally advanced rectal cancer, PD-1/PD-L1 inhibitors combined with neoadjuvant chemoradiotherapy have shown promising response rates and acceptable toxicity profiles. PD-1/PD-L1 inhibitors combined with neoadjuvant chemoradiotherapy hence has a positive outcome even in MSS LARC patients.

**Systematic Review Registration:**

https://www.crd.york.ac.uk/prospero/#myprospero, identifier CRD42023465380.

## 1 Introduction

Neoadjuvant chemoradiotherapy (nCRT) followed by total mesorectal excision (TME) and then adjuvant therapy is considered the standard treatment model for locally advanced rectal cancer (LARC), it has been established that the application of conventional chemoradiotherapy (CRT) followed by radical surgical intervention can yield rates of pathological complete response (pCR) ranging from 10% to 15% (1, 2). However, the overall survival benefits of these preoperative treatment models are limited, with low anal preservation rates and high rates of distant metastasis (3).

In recent years, immune checkpoint inhibitors (ICIs) have emerged as effective treatments for a variety of cancers. KEYNOTE-177 results showed that immunotherapy significantly improved the prognosis of microsatellite instability-high (MSI-H)/deficient mismatch repair(dMMR) advanced colorectal cancer(CRC), and the FDA has approved the pembrolizumab as a standard first-line treatment for MSI-H advanced CRC (4). Although dMMR/MSI-H cancers represent less than 5% of metastatic CRC patients and 10% of early-stage CRC patients, the vast majority of patients have mismatch repair proficient (pMMR) or microsatellite stable (MSS) CRC, which belong to “cold tumors” that are not sensitive to immunotherapy (5, 6). These patients do not appear to benefit from immunotherapy alone (5). However, the NICHE study showed a favorable response to neoadjuvant nivolumab plus ipilimumab in 30% of patients with pMMR/MSS and all patients with dMMR/MSI-H colorectal cancer (7). The study firstly demonstrated the potential benefits of immune checkpoint inhibition in pMMR/MSS CRC. Therefore, many studies have adopted a regimen of nCRT combined with immunotherapy to improve the efficacy of neoadjuvant treatment in LARC patients.

Preclinical research, radiation therapy can cause the immune system to attack tumor cells, release tumor neoantigens, activate antitumor T cells, cause tumor-infiltrating T cells to aggregate, and upregulate PD-L1 expression in tumor tissues. The concomitant application of immunotherapy and radiation therapy could potentiate effectiveness (8, 9). These lay the theoretical foundation for the combination of immunotherapy and radiation, and propose a new treatment for LARC. Therefore, this systematic review evaluated the efficacy and safety of nCRT combined with immunotherapy for LARC patients. Further the pCR rates among different MMR/MSI states (dMMR/MSI-H, pMMR/MSS), different radiotherapy modes (long-course radiotherapy, short-course radiotherapy), different sequence of nCRT combined with immunotherapy (concurrent immunotherapy, sequential immunotherapy) and different immune checkpoint inhibitors (PD-1 inhibitor, PD-1 inhibitor) were compared in the subgroup analysis, in order to find the optimal combination therapy strategy for neoadjuvant therapy of LARC.

## 2 Methods

### 2.1 Search strategy

We conducted comprehensive searches of several online databases from inception to September 2023, which included PubMed, Embase, Cochrane Library, ClinicalTrials.gov, ASCO, and ESMO. Additional electronic searches were performed by conducting thorough investigations into the proceedings of two prominent international conferences, namely, the American Society of Clinical Oncology Annual Meeting and the European Society of Medical Oncology. This was done with consideration for the potential existence of unpublished research findings. The search terms used included “rectal cancer,” “neoadjuvant immunotherapy,” “PD-1,” “PD-L1,” and “neoadjuvant therapy.” Additionally, references from original research studies and literature reviews were also searched in order to ensure that no relevant study was overlooked.

### 2.2 Inclusion and exclusion criteria

The inclusion criteria were as follows: (1) studies involving patients with locally resectable advanced rectal cancer; (2) the primary focus is neoadjuvant chemoradiotherapy involving PD-1/PD-L1 immunosuppressants; and (3) the primary outcomes, including pathologic complete response (pCR), major pathological response (MPR), adverse events (AEs), and the rate of anal preservation, were either directly accessible or indirectly attainable in the study.

The exclusion criteria were as follows: (1) the tumor is metastatic or unresectable; (2) neoadjuvant immunotherapy was not performed; (3) studies without usable data; (4) article type: review, comment, case report, and cell or animal study.

### 2.3 Quality assessment

Most of the included trials were single-arm studies. Consequently, we evaluated the quality of the research using the methodological index for non-randomized studies (MINORS). Studies were classified as poor (0–12), or good (13–16) based on MINORS scores, in that order. Any disagreements were settled by agreement.

### 2.4 Data extraction

Two investigators retrieved the pertinent data from the eligible studies and noted the first author, the year of publication, the number of patients, the interventions, and the pCR rate of the included studies.

### 2.5 Statistical analysis

STATA/SE version 15.1 was used for all statistical analyses. A random-effect model was employed, and heterogeneity between studies was categorized as low (I^2^ < 50%) or high (I^2^ > 50%) using the Cochran Q chi-square test and I^2^ statistics. Sensitivity analysis was conducted by sequentially excluding studies contributing to high heterogeneity in pooled data.

## 3 Results

### 3.1 Study selection and characteristics

**Figure 1:**
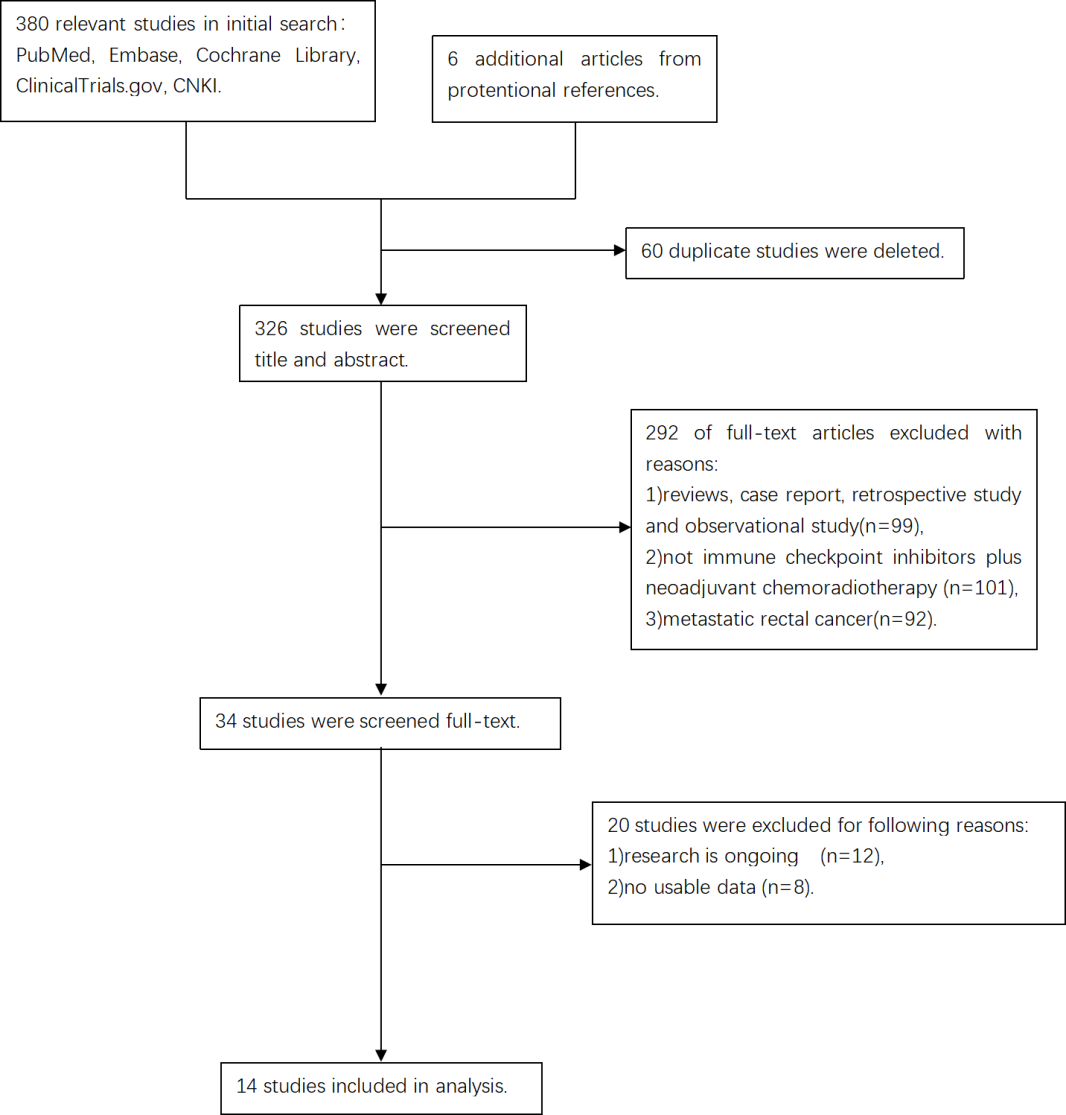
The flow diagram of this meta-analysis.

As seen in **Figure 1**, following the screening of titles and abstracts, elimination of duplicates, and full-text review, 14 studies were ultimately included. The MINORS score system rated fourteen studies as good. **Table 1** summarizes these details.

**Table 1:** Characteristic of included studies.

Study	N	pMMR/MSS	Intervention	pCR (%)	MPR (%)	MINORS score
(10)	101	NA	LCRT(50.4Gy)+Avelumab→TME	23	62	13
(11)	30	28	SCRT(25Gy)→CAPOX+Camrelizumab→TME	48	67	13
(12)	95/90	NA	PA : FOLFOX→LCRT(50.4Gy)+Pembrolizumab→TME	32	NA	15
(13)	60	NA	LCRT(50.4Gy)→Durvalumab→TME	33	58	14
(14)	49	NA	SCRT(25Gy)→FOLFOX+Avelumab→TME	38	68	13
(15)	24	24	CapeOx+Sintilimab→LCRT(50.6Gy)→CapeOx→ W&W/TME	30	20	13
(16)	44	39	LCRT(50.4Gy)→Nivolumab→TME	33	40	13
(17)	25	25	CapeOx+camrelizumab→Cape/LCRT(45Gy)→ CapeOx→TME	33	33	13
(18)	18	18	Cape+LCRT(50Gy)+PD-1→Cape/XELOX+PD-1→ W&W/TME	18	55	13
(19)	45	45	LCRT→Durvalumab→TME	22	NA	13
(20)	62	59	Consolidation : SCRT(25Gy)→CAPOX+toripalimab→W&W/TME Induction : CAPOX+toripalimab→SCRT(25Gy)→ CAPOX+toripalimab→W&W/TME	56	69	13
(21)	39	34	5-Fu+LCRT(45-50Gy)+atezolizumab→TME	27	NA	13
(22)	21	21	SCRT(25Gy)→envafolimab+CAPEOX→TME	67	100	13
(23)	26	26	Cape/LCRT(50Gy)+Tislelizumab→TME	55	27	13

LCRT long−course radiotherapy, SCRT short−course radiotherapy, TME total mesorectal excision, W&W Watch and wait, pCR pathologic complete response, MPR major pathologic response, MINORS methodological index for non-randomized studies.

### 3.2 Efficacy

#### 3.2.1 Tumor response

**Figure 2:**
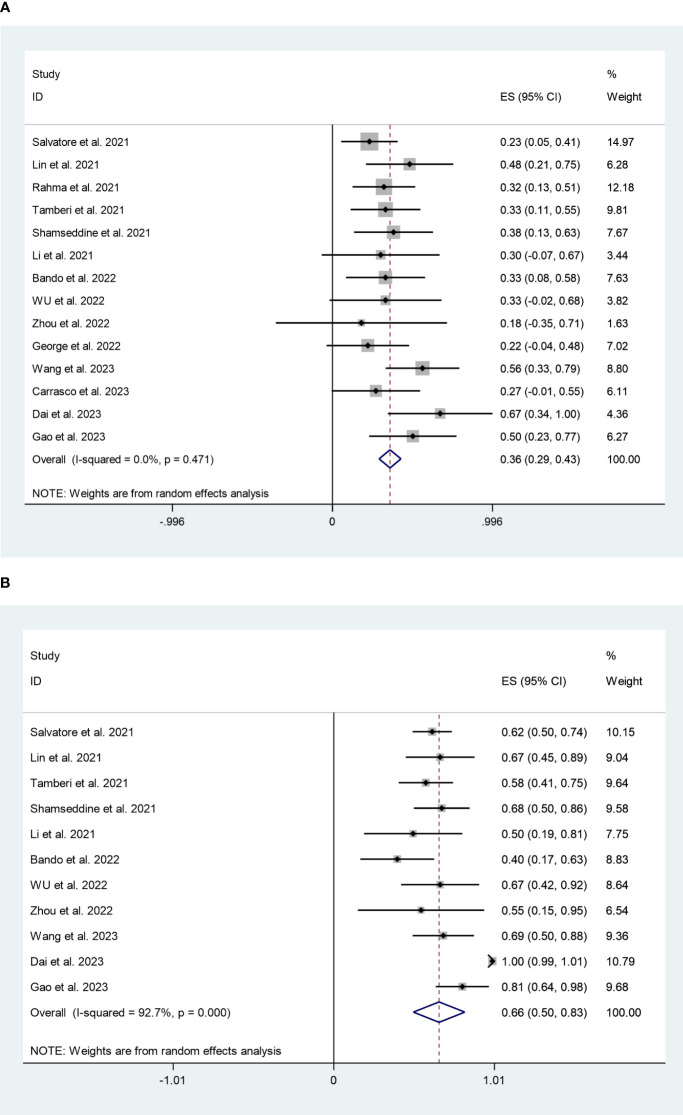
**(A)** Forest plots of pathological complete response rate. **(B)** Forest plots of major pathological response rate.

We analyzed pCR data from 14 studies involving 533 patients. The pooled pCR rate was 36% (95%CI:29%,43%) with minimal heterogeneity (I^2^ = 0%, P=0.471) (**Figure 2A**). A total of eleven studies reported on 382 patients in the MPR. With high heterogeneity (I^2^ = 92.7%, P=0.000), the pooled MPR rate was 66% (95%CI:50%,83%) (**Figure 2B**). Sensitivity analysis revealed that this meta-analysis’s pooled MPR rate was regarded as constant.

#### 3.2.2 Anal preservation rate

**Figure 3:**
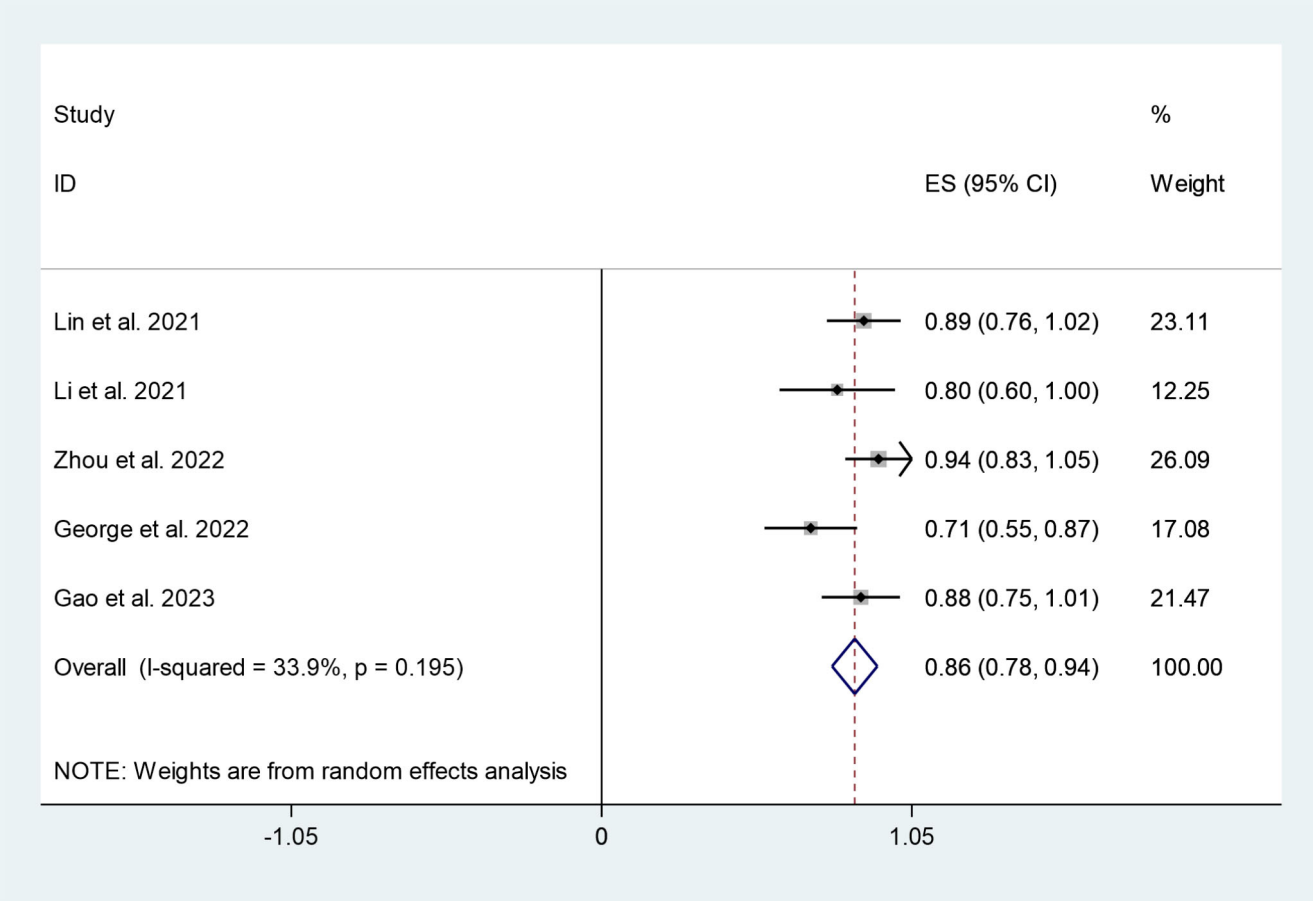
Forest plots of anal preservation rate.

Five studies, including 136 participants, reported an anal preservation rate of 86% (95%CI:78%,94%), although with minimal heterogeneity (I^2^ = 33.9%, P=0.195) (**Figure 3**).

### 3.3 Safety

**Figure 4:**
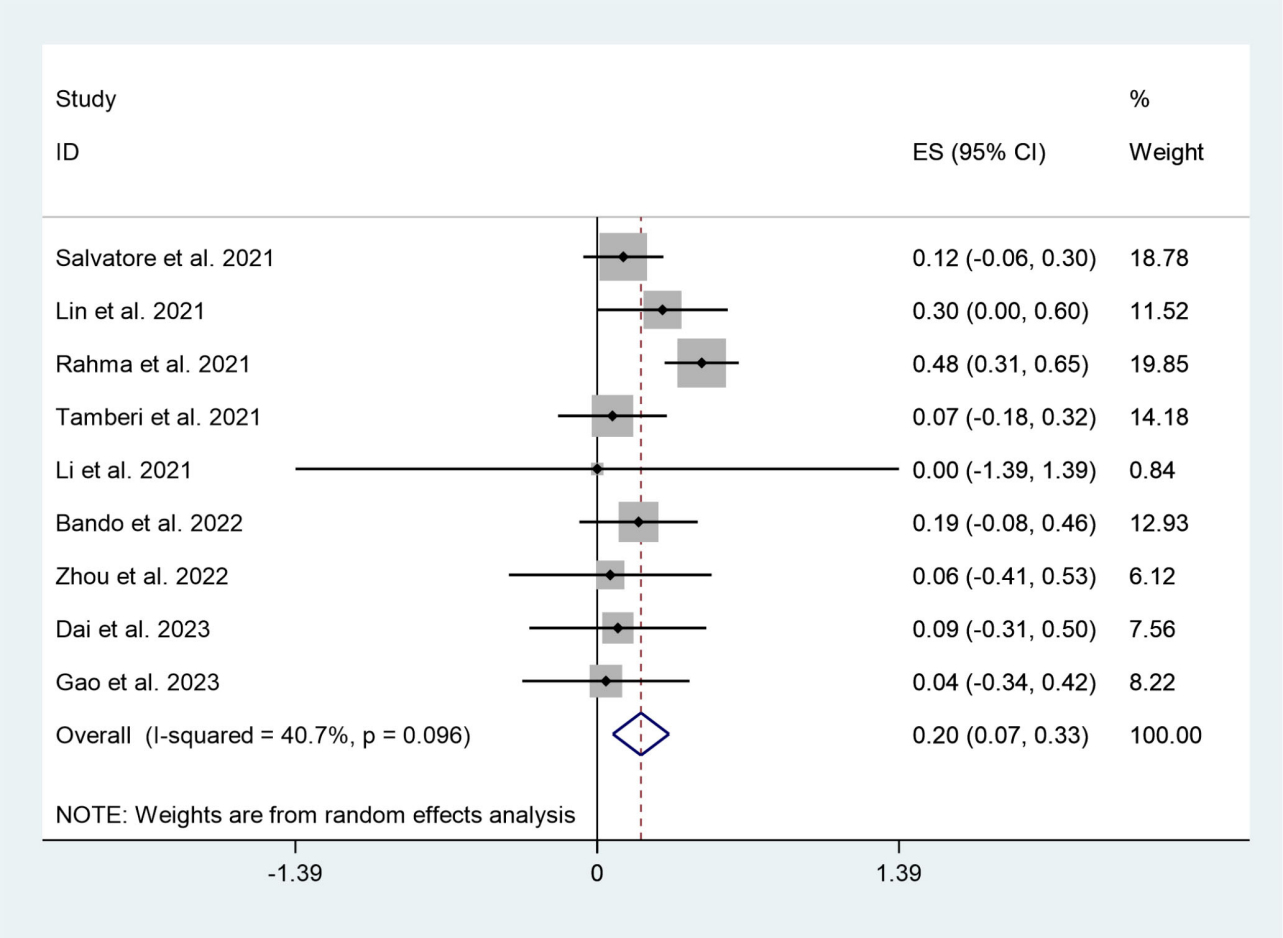
Forest plots of grade≥3 AEs.

There was enough data from nine trials involving 377 patients to analyze the adverse effects grade ≥3 of PD-1/PD-L1 inhibitors plus nCRT; the pooled rate was 20% (95%CI:7%,33%) with minimal heterogeneity (I^2^ = 40.7%, P=0.096) (**Figure 4**).

### 3.4 Subgroup analysis

#### 3.4.1 Subgroup based on mismatch repair status

**Figure 5:**
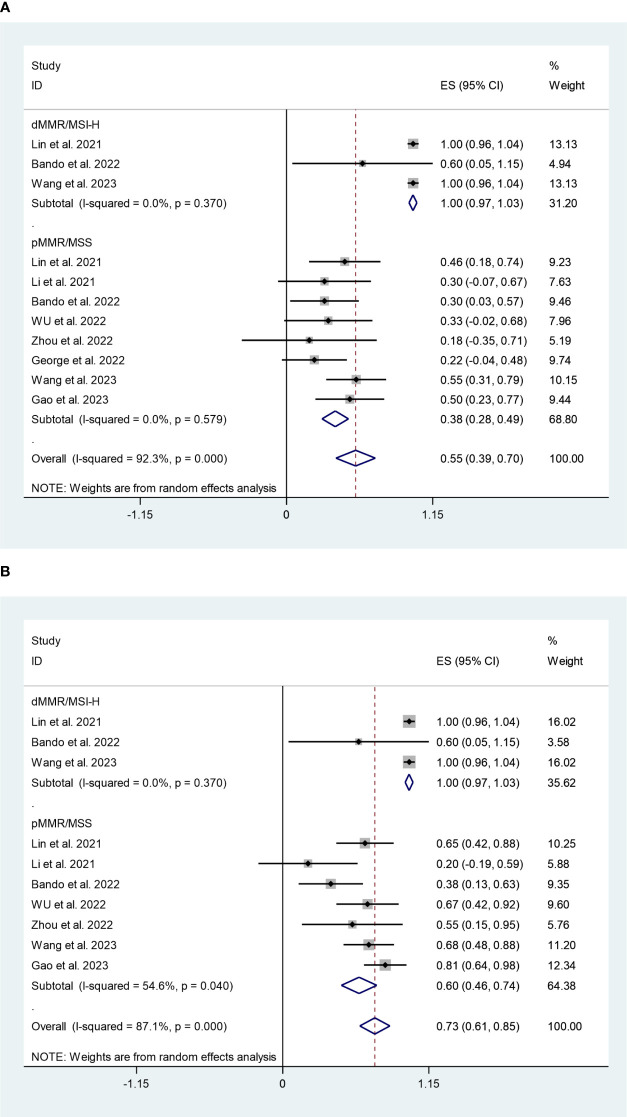
**(A)** Forest plots of mismatch repair status based on pathological complete response rate. **(B)** Forest plots of mismatch repair status based on major pathological response rate.

In dMMR/MSI-H subgroup, the pooled pCR rate was 100% (95%CI:97%,103%) with negligible heterogeneity (I^2^ = 0%, P=0.370) (**Figure 5A**). While, the pMMR/MSS subgroup had a pooled pCR rate of 38% (95%CI:28%,49%) with negligible heterogeneity (I^2^ = 0%, P=0.579) (**Figure 5A**). Additionally, as shown in **Figure 5B**, the subgroup analysis revealed that the pooled MPR rates for the dMMR/MSI-H and pMMR/MSS subgroups were 100% (95%CI:97%,103%, I^2^ = 0%, P=0.370) and 60% (95%CI:46%,74%, I^2^ = 54.6%, P=0.040). Sensitivity analysis revealed that this meta-analysis’s pooled MPR rate was regarded as constant.

#### 3.4.2 Subgroup based on radiotherapy strategies

**Figure 6:**
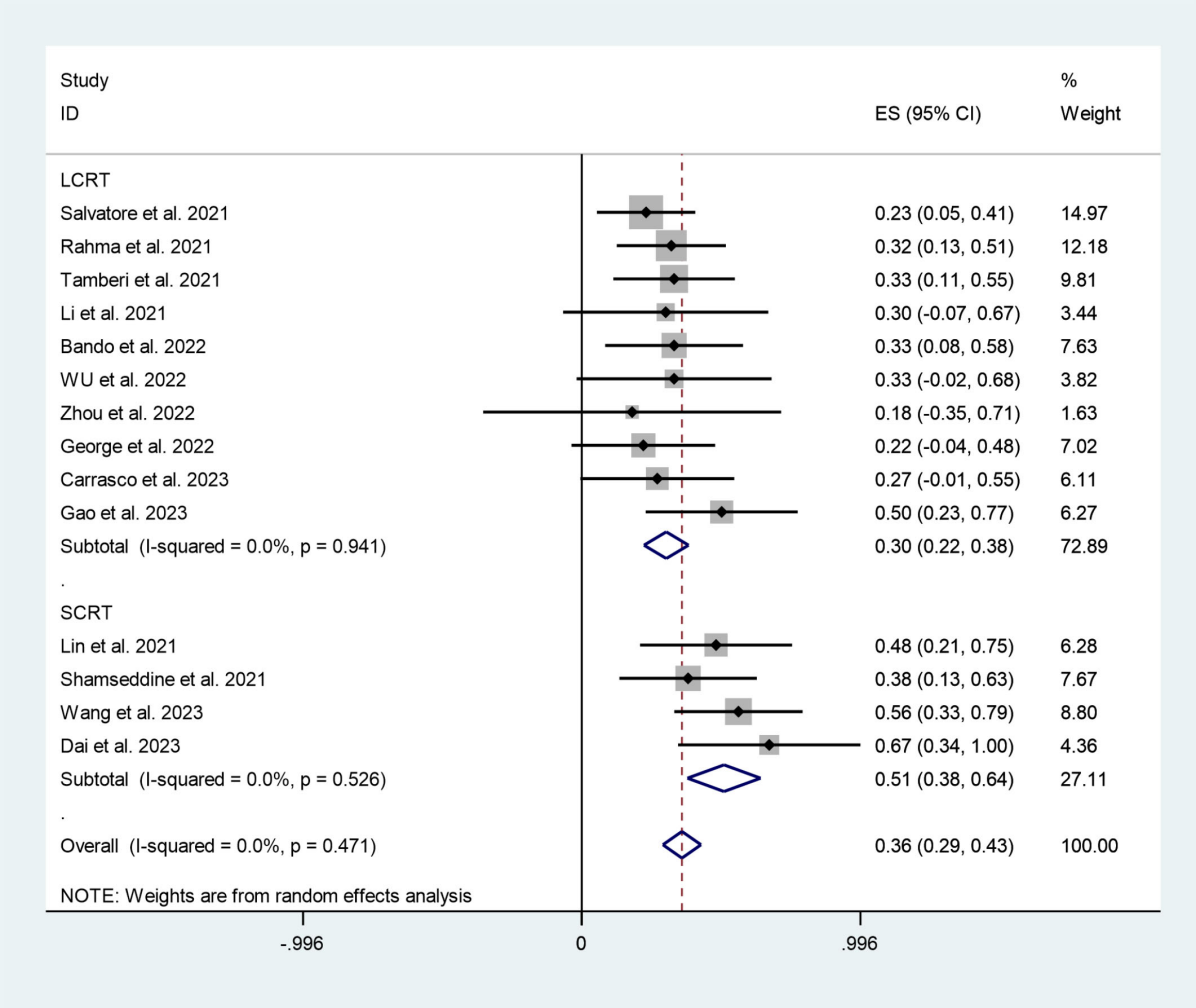
Forest plots of radiotherapy strategies based on pathological complete response rate.

The subgroup analysis revealed that the combined pCR rates for the long-course radiotherapy (LCRT) and short-course radiotherapy (SCRT) subgroups were 30% (95%CI:22%,38%, I^2^ = 0%, P=0.941), 51% (95%CI:38%,64%, I^2^ = 0%, P=0.526) (**Figure 6**).

#### 3.4.3 Subgroup based on neoadjuvant therapy strategies

**Figure 7:**
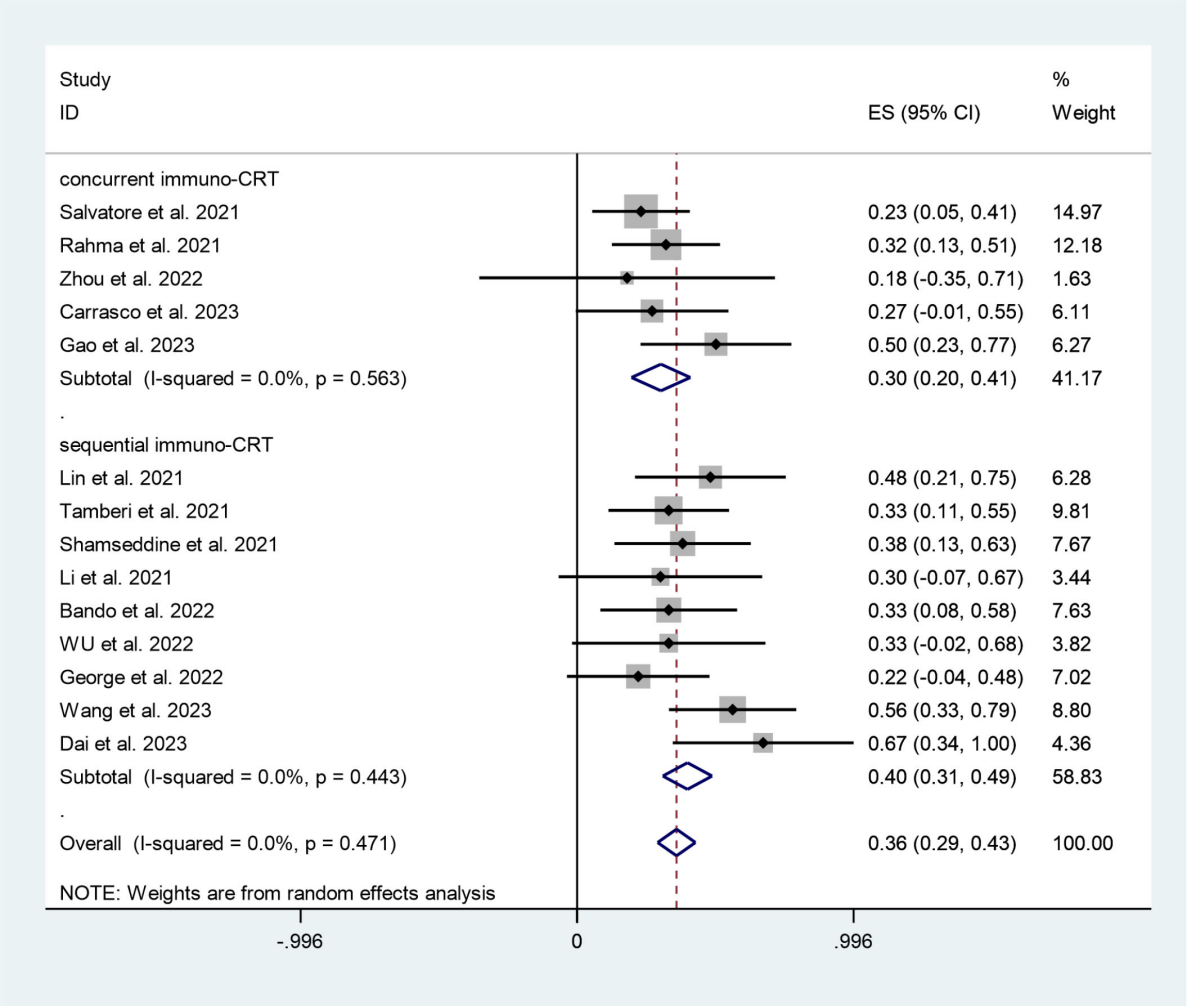
Forest plots of neoadjuvant therapy model based on pathological complete response rate.

The concurrent and sequential immuno-CRT subgroups had pCR rates of 30% (95%CI:20%,41%, I^2^ = 0%, P=0.563) and 40% (95%CI:31%,49%, I^2^ = 0%, P=0.443) respectively, according to the subgroup analysis (**Figure 7**).

#### 3.4.4 Subgroup based on PD-1/PD-L1 inhibitors

**Figure 8:**
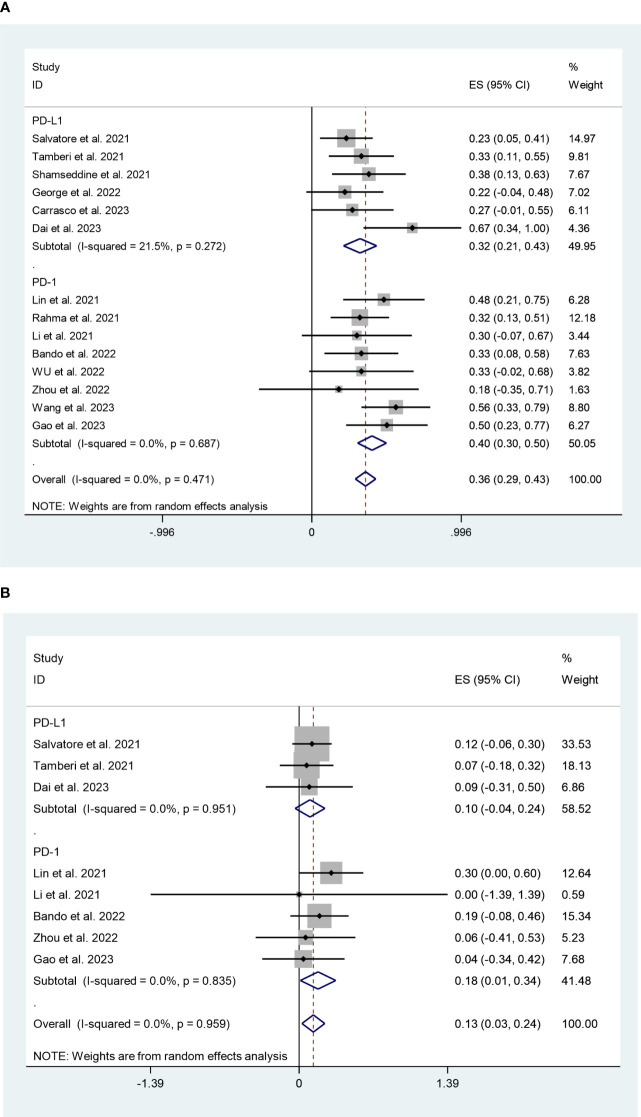
**(A)** Forest plots of PD-1/PD-L1 inhibitors based on pathological complete response rate. **(B)** Forest plots of PD-1/PD-L1 inhibitors based on grade ≥3 effects.

The PD-L1 and PD-1 inhibitors subgroups had pCR rates of 32% (95%CI:21%,43%, I^2^ = 21.5%, P=0.272) and 40% (95%CI:30%,50%, I^2^ = 0%, P=0.687) respectively, according to the subgroup analysis (**Figure 8A**). In terms of safety, the adverse effects grade ≥3 of PD-L1 and PD-1 inhibitors were 10% (95%CI:-4%,24%, I^2^ = 0%, P=0.951) and 18% (95%CI:1%,34%, I^2^ = 0%, P=0.835), respectively (**Figure 8B**).

### 3.5 Publication bias

Analysis using the Egger and Begg tests revealed no evidence of publication bias (P=0.522 and 0.661, respectively).

## 4 Discussion

To our knowledge, this study is one of the earliest report to investigate the safety and efficacy of nCRT when combined with PD-1/PD-L1 inhibitors in patients with locally advanced rectal cancer. Positive outcomes and tolerance to the neoadjuvant PD-1/PD-L1 inhibitors treatment were shown by the results. This systematic review revealed that 36% and 66% of LARC patients treated with nCRT combined with PD-1/PD-L1 inhibitors achieved pCR and MPR, respectively. In comparison, the pCR rate for traditional neoadjuvant chemoradiotherapy is approximately 15% (1), underscoring the potential benefits of adding PD-1/PD-L1 inhibitors. Furthermore, the systematic review showed that this therapeutic method did not significantly raise the incidence of major adverse events (AEs); the pooled anal preservation rate was 86%, and the grade ≥3 toxicity was 20%. Consequently, the combination of PD-1/PD-L1 inhibitors and nCRT emerges as a promising therapeutic strategy that warrants further investigation.

Around 6% of rectal adenocarcinomas are mismatch-repair deficient (24, 25). In the case of individuals diagnosed with metastatic colorectal cancer with dMMR, the utilization of immune checkpoint inhibitors as a first-line treatment had exhibited remarkable effectiveness. This is evidenced by objective response rates falling within the range of 33% to 55%, substantial and clinically significant sustainability of treatment response, and an extension of the overall survival period (26, 27). According to Zhang et al., two MSI-H patients with LARC who received nivolumab as a neoadjuvant therapy saw complete response (CR) (28). Furthermore, the NICHE study explored a combination therapy of nivolumab and the ipilimumab in early-stage (II-III) CRC patients, showed 100% pathological response in patients with dMMR tumors. Allowing immunotherapy for LARC to be initiated in the preoperative stage (7). Our study also revealed that the MSI-H/dMMR LARC subgroup had a pooled pCR rate of 100%,suggesting that nCRT combined with immunotherapy leads to superior outcomes in patients with dMMR/MSI-H LARC.

A majority of LARC patients had MSS tumors, however, MSS LARC patients, had no responses to immunotherapy in Keynote-016 and Keynote-028, the single-agent ICI provided very little benefit to MSS tumors (5, 29). The 30 patients in the pMMR/MSI-L cohort responded pathologically to nivolumab+ipilimumab in the NICHE trial by 30%, with a 10% pCR rate, marking the first instance of immune checkpoint inhibition efficacy in pMMR/MSS patients (7). As a result, research had begun into combinations treatment that enhance the effectiveness of immunologic therapy in this subtype (30, 31). Currently researchers are primarily focused on how ICIs and nCRT together can boost efficacy in MSS LARC patients by overcoming the “immune-cold” tumor microenvironment.

Preclinical research, radiation therapy could cause the immune system to attack tumor cells, release tumor neoantigens, activate antitumor T cells, cause tumor-infiltrating T cells to aggregate, and upregulate PD-L1 expression in tumor tissues (32, 33). The concomitant application of immunotherapy and radiation therapy appeared to potentiate effectiveness (34, 35). The observation of distant effects in clinical research involving immunotherapy and radiotherapy was seen as compelling evidence that the latter enhances the body’s immune response against tumors. These form the theoretical foundation for the combination of immunotherapy and radiation (36, 37).

We examined the effectiveness of nCRT plus PD-1/PD-L1 inhibitors for pMMR/MSS patients with LARC based on the aforementioned research and theories, the pCR and MPR rates were 38% and 60%, respectively. Therefore, radiation therapy is anticipated to make MSS LARC patients more sensitive to immunotherapy. Future research should focus on studies can be conducted to find markers that can clearly predict therapeutic efficacy.

Positive PD-L1 expression and high TMB were reliable indicators of neoadjuvant effectiveness, according to the VOLTAGE-A research (16). Similarly Lin et al. found that compared to patients with CPS <1/TMB <10, the pCR of patients with PD-L1 CPS ≥1/TMB ≥10 was substantially greater (11). Neoadjuvant chemoradiotherapy combined with immunotherapy had significantly improved the rate of pathological remission in LARC patients, however, special attention was still needed for LARC patients with high risk of disease progression after surgery. Therefore, exploring the effect of the addition of immunotherapy on tumor regression grade(TRG) and searching more reliable clinico-pathological biomarkers could improve the evaluation performance of postoperative prognosis and assist in planning postoperative treatment. Sert et al. used the TRG system and neoadjuvant rectal score(NARS) to assess the postoperative pathological response of LARC patients receiving nCRT, compared the value of TRG system and NARS in determining the prognosis, and found that the NARS may be a more favorable tool for predicting overall survival(OS) and metastasis free survival(MFS) (38). In addition, the watch and wait (W&W) strategy may be safe for LARC patients in complete clinical remission (cCR), it was challenging to accurately select these patients, therefore minimizing diagnostic differences among endoscopists was crucial. Wang et al. found that the deep learning algorithms of endoscopic images could accurately assessed the response of LARC to neoadjuvant therapy, and help to exactly select the best candidates for W&W strategies (39). Although the clinical strategy of nCRT combined with immunotherapy had great potential, there were still many areas that need to be improved. Artificial intelligence and its subtypes, especially deep learning(DL) played an increasing role in the diagnosis and prognosis of LARC, DL protocols seemed to help physicians optimize the strategy of neoadjuvant treatment of LARC by improving diagnostic accuracy, increasing clinician experience, and minimizing diagnostic subjectivity among physicians (40, 41).

It’s possible that the proinflammatory effects of RT and ICIs will produce intolerable toxicity. According to a meta-analysis, 68.3% of grade 3 or above adverse events were treatment-related side effects for immunotherapy plus chemotherapy. Additionally, the rate of grade 3 or higher adverse events in radiotherapy combinations with immunotherapy was 12.4% (42). Our finding demonstrated that PD-1/PD-L1 inhibitors plus nCRT did not increase the occurrence of significant adverse events. The pooled rates of ≥3 AEs were 20%. Moreover the surgical site infection(SSI) was one of the most common adverse events following rectal cancer procedures, among which sepsis was a life-threatening complication. Mulita et al. found that in rectal cancer patients the incidence of SSI was 21.8%, the postoperative sepsis was 12.77%, and advanced age, diabetes, cardiovascular disease and American Society of Anesthesiologists(ASA) class >2 are important factors affecting the occurrence of SSI and postoperative sepsis (43, 44). Interestingly, the studies included in this systematic review showed that immunotherapy combined with nCRT did not increase the incidence of SSI and postoperative sepsis, and there were no documented surgery-related deaths (15–17, 20, 23). This allows us to posit that PD-1/PD-L1 inhibitors and CRT combination therapy has a favorable safety profile.

In evaluating radiotherapy dosing and segmentation, previous studies have shown that short-course radiotherapy followed by double-drug chemotherapy holds advantages over long-course concurrent chemoradiotherapy in terms of tumor regression and overall survival (2, 45). This raises the question of whether its combination with immunotherapy could further enhance these outcomes. Our findings show that, while the pCR rates for PD-1/PD-L1 inhibitors with long-course radiotherapy(LCRT) and short-course radiotherapy(SCRT) were 30% and 51%, respectively. The aforementioned data demonstrated compared to LCRT, SCRT with immunotherapy presenting notably higher pCR rates. The study of different fractionation modes combined with immunotherapy in colorectal cancer found that the results of radiotherapy combined with immunosuppressants at three biologic effect doses of 16.4Gy×1, 8Gy×3 and 2Gy×18 showed that 8Gy×3 was the best segmentation mode, which significantly increased the number of tumor-infiltrating lymphocytes and the expression of PD-L1 and TIGIT, and could most effectively control tumor development (90% complete response) after combining ICI (46). In the same animal model, Morisada et al. showed that short-course hypofractionation could achieve better local and ectopic anti-tumor effects compared with conventional fractionation, such as increasing tumor-specific CD8+ T cells in draining lymph nodes, and concluded that ICI combined with hypofractionated radiotherapy was more effective than conventional fractionation to break immune tolerance and achieve stronger anti-tumor immune effects (47). In addition, it has been found that short-course hypofractionated radiotherapy can effectively inhibit tumor growth and recruit T lymphocytes in draining lymph nodes, maintain low levels of suppressor T cells and high levels of effector T cells, and at the same time, short-course hypofractionated radiotherapy can increase the levels of IL-8, IL-6, TNFa and other factors, and has a stronger effect of promoting dendritic cell (DC) maturation and activation, thereby increasing the efficacy of immunotherapy (48, 49). At present, most clinical studies use SBRT combined with immunotherapy, with a small number of SBRT fractions and a large single dose, taking into account the effect of tumor local control(LC) and promoting immune function. This study is the first to show that the perioperative SCRT combination with immunochemotherapy for LARC has a higher pCR rate than LCRT.

Our systematic review found higher pCR rates following the sequential (40%) immuno-CRT compared to concurrent immuno-CRT (30%). As for the timing of radiotherapy combined with immunotherapy, from the mechanism of action, it has been suggested that immune checkpoint inhibitors can not only activate killer T cells, but also normalize tumor blood vessels, reduce the state of tumor hypoxia, and increase the possibility of tumor sensitivity to radiotherapy (50). Therefore, immunoinduction followed by radiotherapy may improve the efficacy of radiotherapy, but the use of this model requires screening out the population sensitive to immunotherapy in advance. On the other hand, radiotherapy can activate the body’s immunity through the release of tumor antigen, so radiotherapy sequential immunotherapy may be more promising. However, the exposure of tumor antigen in the human body after release has a certain timeliness, and it will be cleared by the body after too long, which also poses challenges for the timing of immunotherapy intervention. As for which of the above two forms is better, there is no definite answer (51). This is mainly due to the difference in the target of immune checkpoint inhibitors and the difference in the clinical stage of patients. Therefore, it is necessary to explore the most appropriate combination therapy according to the drug and the patient’s own situation.

A meta-analysis showed that PD-1 inhibitors had an OS advantage over PD-L1 inhibitors (HR0.75), regardless of monotherapy (HR0.78) or combination chemotherapy (HR0.68), and PD-1 inhibitors also had better progression-free survival(PFS). The OS advantage of PD-1 versus PD-L1 inhibitors appeared to be more pronounced in combination therapy (HR0.67) than immunomonotherapy (HR0.85) (52). The results of this study showed that the pCR rate of PD-L1 inhibitors and PD-1 inhibitors was respectively 32% and 40%. Chemoradiotherapy can increase the expression of PD-L1 in tumor cells, leading to the synergy of immune drugs and chemoradiotherapy, and additional PD-L1 expression may offset T cell activation through excessive consumption of PD-L1 antibodies, thus causing this difference (53). And with PD-L1 inhibitors, the tumors escaped the anti-tumor immune response via the PD-1/PD-L2 axis. On the other hand, PD-L1 inhibition also played an important role in blocking the interaction between PD-L1 and CD80, so that CD80 preferentially binded to CTLA-4 on the surface of T cells, and the binding of CTLA-4 and CD80 negatively regulated the activation of T lymphocytes, which can only be achieved by PD-L1 antibodies (54). Research by Chen et al. indicated that exosomes in malignant melanoma also express PD-L1, which can directly bind to PD-1 on the surface of T cells and inhibit T cell function, and can also bind to PD-L1 monoclonal antibody.PD-L1 monoclonal antibodies may be neutralized by exosomes PD-L1 before they have a role to play (55). In terms of safety, our study found that PD-1 subgroup(18%) had a higher incidence of adverse events than PD-L1 subgroup(10%), and PD-1/PD-L2 may play an important role in mediating lung immune tolerance. PD mice experiment showed that PD-1inhibitors could undermine PD-1/PD-L2 combined with other partners such as repulsive guidance molecule b (RGMb) the interaction of balance, and result in adverse events (56), which may provide a useful guide for clinicians.

This systematic review has various limitations. First, approximately 50% of the data were included from active studies or abstracted from conferences, resulting in a limited number of patients and incomplete clinical data. Therefore, a large number of research are still needed to confirm the significance of nCRT combined with immunotherapy in LARC patients. Second, heterogeneity is further exacerbated by differences in immunotherapeutic drugs, patient characteristics, and study designs, the high heterogeneity in the pooled MPR rate is a concern, although slight improvement was observed in the subgroup analysis. Third, nCRT combined with immunotherapy is a relatively new treatment modality, up to now only one of the included studies had long-term survival data. Rahma et al. found that nCRT plus pembrolizumab was associated with a statistically significant improvement in 3yr OS compared with nCRT (95% vs 87%, *P*=0.04), but not in DFS. Since only one study reported long-term efficacy, there were insufficient direct comparative data on time-to-event outcomes, such as OS and DFS, in order to evaluate the survival outcomes of patients with LARC who received nCRT combined with immunotherapy. Pending the release of data on OS and DPS in subsequent studies, we will continue to analyze whether this treatment strategy will benefit long-term survival in LARC patients. Fourth, the predominance of single-arm, phase II clinical trials with limited sample sizes in the existing clinical research, which could lead to over- reporting of the efficacy of these neoadjuvant regimens. Despite these limitations, we found that there was increased efficacy when adding PD-1/PD-L1 inhibitors to nCRT for LARC patients, and our findings suggested possible directions for future treatment strategies.

## 5 Conclusion

These data suggested that nCRT combined with immunotherapy can improve MPR rate and pCR rate of pMMR/MSS locally advanced rectal cancer, while patients with dMMR/MSI-H had a higher pathological response rate. Preoperative immunotherapy combined with chemoradiotherapy can improve anal retention in patients with low rectal cancer, without increasing the incidence of adverse events. In addition, short-course radiotherapy, PD-1 inhibitors, and sequential immuno-CRT were associated with higher pCR rates compared to long-course radiotherapy, PD-L1 inhibitors, and concurrent immune CRT. In conclusion, this study provides favorable supporting evidence for neoadjuvant chemoradiotherapy combined with immunotherapy for locally advanced rectal cancer.

## Data availability statement

The original contributions presented in the study are included in the article/**Supplementary Material**. Further inquiries can be directed to the corresponding author.

## Author contributions

LY: Conceptualization, Data curation, Formal analysis, Funding acquisition, Investigation, Methodology, Project administration, Resources, Software, Supervision, Validation, Visualization, Writing – original draft, Writing – review & editing. XC: Data curation, Project administration, Software, Supervision, Visualization, Writing – review & editing. FW: Data curation, Investigation, Methodology, Project administration, Resources, Supervision, Validation, Visualization, Writing – review & editing. ZC: Data curation, Formal analysis, Investigation, Methodology, Project administration, Software, Visualization, Writing – review & editing. LX: Conceptualization, Formal analysis, Investigation, Methodology, Resources, Supervision, Validation, Writing – review & editing. XW: Formal analysis, Investigation, Methodology, Project administration, Visualization, Writing – review & editing. ZL: Conceptualization, Methodology, Project administration, Software, Writing – review & editing. XNL: Writing – review & editing. QY: Data curation, Software, Writing – review & editing. XQL: Conceptualization, Data curation, Formal analysis, Funding acquisition, Investigation, Methodology, Project administration, Resources, Software, Supervision, Validation, Visualization, Writing – review & editing. CG: Conceptualization, Formal analysis, Funding acquisition, Investigation, Methodology, Project administration, Visualization, Writing – review & editing.
