# Supplementary material for: The efficacy and safety of neoadjuvant chemoradiotherapy combined with immunotherapy for locally advanced rectal cancer patients: a systematic review
Source: Front Immunol. 2024 May 15;15:1392499. doi: 10.3389/fimmu.2024.1392499 (PMC11154111; doi:10.3389/fimmu.2024.1392499)
Supplement: Supplementary file 1 [file DataSheet_1.docx]

Sensitivity analysis of major pathological response rate

Sensitivity analysis of grade≥3 AEs

Sensitivity analysis of pMMR/MSS subgroup based on major pathological response rate

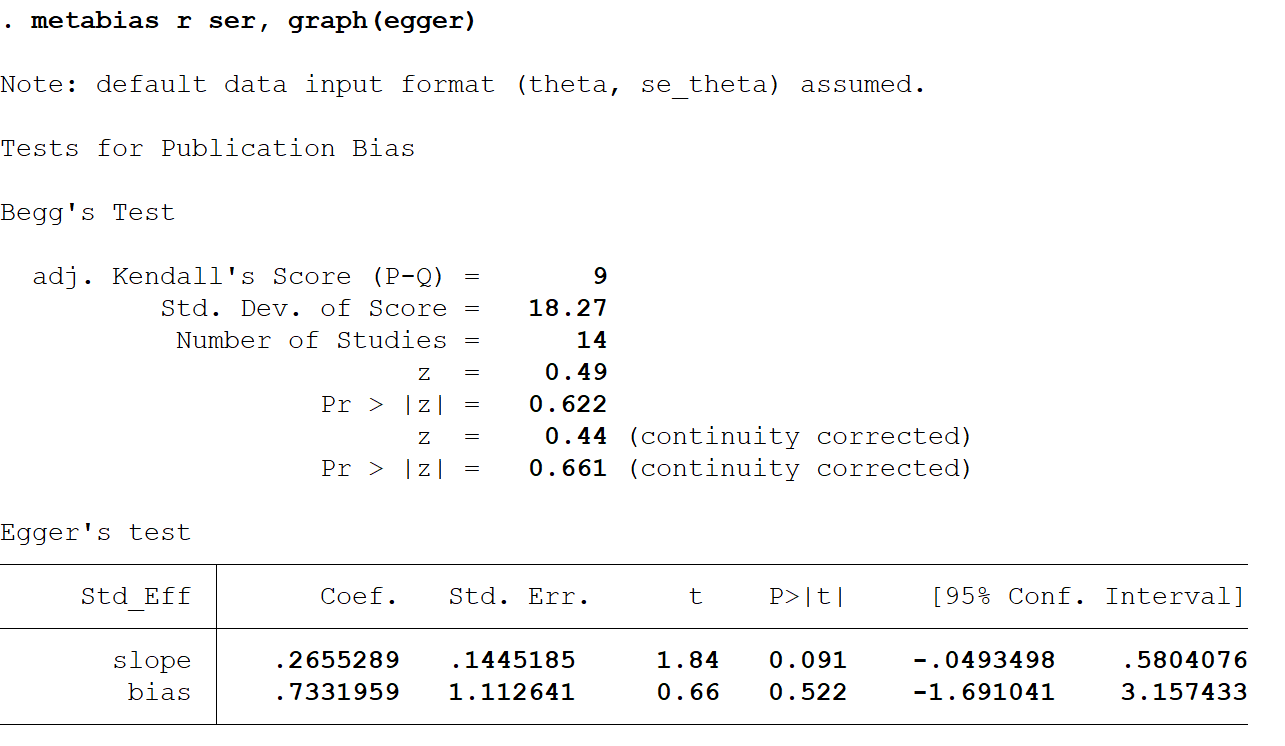


Publication bias

MINORS biased risk assessment

| Study | A | B | C | D | E | F | G | H | score |
| --- | --- | --- | --- | --- | --- | --- | --- | --- | --- |
| (Salvatore et al., 2021) | 2 | 2 | 2 | 2 | 2 | 1 | 1 | 1 | 13 |
| (Lin et al., 2021) | 2 | 2 | 2 | 2 | 2 | 1 | 1 | 1 | 13 |
| (Rahma et al., 2021) | 2 | 2 | 2 | 2 | 2 | 2 | 2 | 1 | 15 |
| (Tamberi et al., 2022) | 2 | 2 | 2 | 2 | 2 | 1 | 1 | 2 | 14 |
| (Shamseddine et al., 2021) | 2 | 2 | 2 | 2 | 2 | 1 | 1 | 1 | 13 |
| (Li et al., 2021) | 2 | 2 | 2 | 2 | 2 | 1 | 1 | 1 | 13 |
| (Bando et al., 2022) | 2 | 2 | 2 | 2 | 2 | 1 | 1 | 1 | 13 |
| (WU et al., 2022) | 2 | 2 | 2 | 2 | 2 | 1 | 1 | 1 | 13 |
| (Zhou et al., 2022) | 2 | 2 | 2 | 2 | 2 | 1 | 1 | 1 | 13 |
| (George et al., 2022) | 2 | 2 | 2 | 2 | 2 | 1 | 1 | 1 | 13 |
| (Wang et al., 2022) | 2 | 2 | 2 | 2 | 2 | 1 | 1 | 1 | 13 |
| (Carrasco et al., 2023) | 2 | 2 | 2 | 2 | 2 | 1 | 1 | 1 | 13 |
| (Dai et al., 2023) | 2 | 2 | 2 | 2 | 2 | 1 | 1 | 1 | 13 |
| (Gao et al., 2023) | 2 | 2 | 2 | 2 | 2 | 1 | 1 | 1 | 13 |

Note:1. A stated aim of the study, 2.Inclusion of consecutive patients, 3. Prospective collection of data, 4. Endpoint appropriate to the study aim, 5. Unbiased evaluation of endpoints, 6.Follow-up period appropriate to the major endpoint, 7.Loss to follow up not exceeding 5%, 8.Prospective calculation of the sample size
